# *CSRNP1* Promotes Apoptosis and Mitochondrial Dysfunction via ROS-Mediated JNK/p38 MAPK Pathway Activation in Hepatocellular Carcinoma

**DOI:** 10.32604/or.2025.068737

**Published:** 2025-12-30

**Authors:** Huihui Shi, Lei Chen, Juan Huang, Xuejing Lin, Lei Huang, Min Tang, Kai Lu, Wenchao Wang, Maoling Zhu

**Affiliations:** 1 Department of Gastroenterology, Yangpu Hospital, School of Medicine, Tongji University, Shanghai, 200090, China; 2 Center for Clinical Research and Translational Medicine, Yangpu Hospital, School of Medicine, Tongji University, Shanghai, 200090, China; 3 Department of Surgical Room, Yangpu Hospital, School of Medicine, Tongji University, Shanghai, 200090, China; 4 Department of General Surgery, Yangpu Hospital, School of Medicine, Tongji University, Shanghai, 200090, China; 5 Department of Hepatic-Biliary-Pancreatic Surgery, Shanghai Fourth People’s Hospital, School of Medicine, Tongji University, Shanghai, 200434, China

**Keywords:** Cysteine/serine-rich nuclear protein 1, c-Jun N-terminal kinase/p38 mitogen-activated protein kinase pathway, hepatocellular carcinoma, reactive oxygen species accumulation, mitochondrial dysfunction

## Abstract

**Background:**

Hepatocellular carcinoma (HCC) is one of the leading causes of cancer-related mortality worldwide. This study aimed to identify key genes involved in HCC development and elucidate their molecular mechanisms, with a particular focus on mitochondrial function and apoptosis.

**Methods:**

Differential expression analyses were performed across three datasets—The Cancer Genome Atlas (TCGA)-Liver Hepatocellular Carcinoma (LIHC), GSE36076, and GSE95698—to identify overlapping differentially expressed genes (DEGs). A prognostic risk model was then constructed. Cysteine/serine-rich nuclear protein 1 (*CSRNP1*) expression levels in HCC cell lines were assessed via western blot (WB) and quantitative reverse transcription polymerase chain reaction (qRT-PCR). The effects of *CSRNP1* knockdown or overexpression on cell proliferation, migration, and apoptosis were evaluated using cell counting-8 (CCK-8) assays, Transwell assays, and flow cytometry. Mitochondrial ultrastructure was examined by transmission electron microscopy, and intracellular and mitochondrial reactive oxygen species (mROS) levels were measured using specific fluorescent probes. WB was used to assess activation of the c-Jun N-terminal kinase (JNK)/p38 mitogen-activated protein kinase (MAPK) pathway, and pathway dependence was examined using the ROS scavenger N-Acetylcysteine (NAC) and the JNK inhibitor SP600125.

**Results:**

A six-gene prognostic model was established, comprising downregulated genes (*NR4A1* and *CSRNP1*) and upregulated genes (*CENPQ*, *YAE1*, *FANCF*, and *POC5*) in HCC. Functional experiments revealed that *CSRNP1* knockdown promoted the proliferation of HCC cells and suppressed their apoptosis. Conversely, *CSRNP1* overexpression impaired mitochondrial integrity, increased both mitochondrial and cytoplasmic ROS levels, and activated the JNK/p38 MAPK pathway. Notably, treatment with NAC or SP600125 attenuated *CSRNP1*-induced MAPK activation and apoptosis.

**Conclusion:**

*CSRNP1* is a novel prognostic biomarker and tumor suppressor in HCC. It exerts anti-tumor effects by inducing oxidative stress and activating the JNK/p38 MAPK pathway in a ROS-dependent manner. These findings suggest that *CSRNP1* may serve as a potential therapeutic target in the management of HCC.

## 1 Introduction

Hepatocellular carcinoma (HCC) ranks among the top three causes of cancer-related mortality worldwide [1]. Current epidemiological projections estimate that the global incidence of liver cancer will rise from approximately 905,347 cases in 2020 to 1,392,474 by 2040 [2]. Extensive research has established that individuals with chronic liver diseases—particularly those with hepatitis B virus (HBV) or hepatitis C virus (HCV) infections, alcoholic liver disease, or non-alcoholic fatty liver disease (NAFLD)—are at a significantly increased risk of developing HCC [3,4]. In Asia and Africa, hepatitis virus infection remains the predominant etiological factor [5]. The widespread implementation of antiviral therapies has led to a decline in HCC incidence associated with viral hepatitis in several countries [6]. Current treatment options for HCC include surgical resection, transarterial chemoembolization, local radiofrequency ablation, and molecular-targeted therapies [7,8]. Clinical advancements have highlighted the importance of early detection, routine surveillance, and long-term follow-up in improving patient outcomes. Recent evidence also suggests that mitochondrial metabolic dysfunction is closely linked to the development and metastasis of HCC [9]. Disruptions in oxidative phosphorylation (OXPHOS) and excessive production of reactive oxygen species (ROS) compromise mitochondrial homeostasis, contributing to tumor progression [10]. Therefore, elucidating the underlying molecular mechanisms and identifying robust biomarkers for HCC are critical for improving diagnostic accuracy and developing more effective therapeutic strategies.

Cysteine/serine-rich nuclear protein 1 (*CSRNP1*), also known as *AXIN1 upregulated 1* (*AXUD1*), encodes a nuclear protein that belongs to the CSRNP gene family [11]. *CSRNP1* contains cysteine- and serine-rich regions, along with multiple nuclear localization signals and transcriptional activation domains, suggesting a regulatory role in transcription(S), along with numerous atomic localization signals and transcriptional activation domains, suggesting its potential role in transcriptional regulation [12]. Zhang et al. reported that low expression of *CSRNP1* and *CSRNP3* is associated with poor overall survival in clear cell renal cell carcinoma (ccRCC), and a prognostic model incorporating these genes was developed [13]. In non-small cell lung cancer, Xu et al. found that *CSRNP1* is downregulated, and its silencing promotes cancer cell malignant phenotype, possibly through the AKT/MDM2/p53 pathway, indicating a tumor-suppressive role [14]. In liver ischemia-reperfusion injury (HIRI), *CSRNP1* expression is significantly elevated and correlates with activation of the MAPK pathway. Notably, *CSRNP1* knockdown alleviates liver injury, reduces inflammation, and inhibits apoptosis in both *in vitro* and *in vivo* models [15]. Although recent studies have begun to investigate *CSRNP1* in liver pathologies, including HCC, direct mechanistic insights remain limited. For example, Ishiguro et al. showed that, in contrast to nearby non-tumor liver tissue, CSRNP1 expression is markedly downregulated in HCC tissues [16]. Similarly, Xu et al. used transcriptome profiling to suggest that *CSRNP1* may serve as a prognostic and progression-related biomarker in HCC [17]. Collectively, these findings point to a potential tumor-suppressive function for *CSRNP1* in HCC. However, research into the role of *CSRNP1* in cancer, particularly HCC, remains in its early stages and warrants further investigation.

The c-Jun N-terminal kinase (JNK) and p38 mitogen-activated protein kinase (MAPK) pathways are critical regulators of various biological processes, including cell proliferation, differentiation, stress response, and apoptosis [18]. As members of the MAPK family, both JNK and p38 are primarily activated in response to cellular stress [19]. These kinases play dual roles in cancer, and numerous studies have explored their involvement in HCC. For example, Chen et al. demonstrated that serum deprivation response protein (SDPR) promotes apoptosis in HCC cells by activating the apoptosis signal-regulating kinase 1 (ASK1)–JNK/p38 MAPK pathway, thereby inhibiting proliferation, invasion, and migration [20]. Yang et al. demonstrated that neurotrophin-3 (NTF3) induces apoptosis in HCC cells through JNK and p38 MAPK activation mediated by the p75 neurotrophin receptor [21]. Deng et al. found that Carfilzomib (CFZ) inhibits HCC cells’ growth while inducing ROS generation, endoplasmic reticulum stress, and JNK/p38 MAPK activation [22]. In addition, Sarg et al. reported that the p38 MAPK pathway modulates mitochondrial responses to oxidative stress and apoptosis triggered by natural compounds or chemotherapeutic agents, thereby offering potential therapeutic opportunities for cancer and metabolic disorders [23]. Collectively, these studies underscore the significance of the JNK/p38 MAPK pathway in HCC pathogenesis and treatment.

HCC is a highly aggressive malignancy driven by complex signaling networks that regulate proliferation, apoptosis, and metastasis. Among these, the JNK/p38 MAPK cascade plays a crucial role in mediating stress responses and determining cell fate. However, the interplay between this pathway and specific tumor suppressor genes remains poorly understood. *CSRNP1* has emerged as a potential tumor suppressor that may influence stress-related signaling; however, its regulatory relationship with the JNK/p38 MAPK pathway in HCC remains to be fully elucidated. Investigating the role of *CSRNP1* in modulating this pathway could provide novel insights into HCC pathophysiology and uncover new targets for therapeutic intervention.

## 2 Materials and Methods

### 2.1 Identification of Differentially Expressed Genes (DEGs) in HCC via Integrated Analysis of Public Datasets

Three publicly accessible HCC datasets provided gene expression profiles. The Clinical Bioinformatics Database (https://www.bioinfo-zs.com) provided the Cancer Genome Atlas (TCGA)-Liver Hepatocellular Carcinoma (LIHC) dataset, which included 371 tumor samples and 50 adjacent normal liver tissues. The GSE36076 dataset includes 10 tumor samples and 10 adjacent non-tumor tissue samples. Additionally, the GSE95698 dataset included 3 tumor and 6 adjacent non-tumor tissue samples. Differential expression analysis was performed independently for each dataset using fold change (FC) thresholds to define DEGs. Substantially upregulated genes were considered as having FC above 2 and *p* less than 0.05, whilst substantially downregulated genes were regarded as having FC less than 0.5 and *P* less than 0.05. Volcano plots were created using the R package “ggplot2” (version 3.6.3). To identify DEGs across datasets, Venn diagram analysis was conducted utilizing the Venn online graph tool (https://bioinformatics.psb.ugent.be/webtools/Venn/ (accessed on 01 September 2025)). Overlapping upregulated and downregulated DEGs among the Cancer Genome Atlas-Liver Hepatocellular Carcinoma (TCGA-LIHC), GSE36076, and GSE95698 datasets were determined and selected for further investigation as candidate genes potentially involved in HCC pathogenesis.

### 2.2 Construction and Validation of a Prognostic Risk Model Using Least Absolute Shrinkage and Selection Operator (LASSO) Cox Regression in HCC

The “glmnet” package (version 4.1-2) in R (version 4.2.2) was used to conduct LASSO Cox regression analysis to create a predictive gene signature for HCC. Nine potential predictive genes, previously identified through integrated differential expression analysis, were subjected to LASSO regression to determine the most robust prognostic features. The ideal lambda (λ) value that avoided overfitting and reduced partial likelihood deviation was found using ten-fold cross-validation. The following formula was utilized to determine each patient’s prognostic risk score based on the chosen gene set: ∖text{RiskScore}=0.3756∖cdot∖text{CENPQ}+0.1956∖cdot∖text{YAE1}+0.1556∖cdot∖text{POC5}+0.0013∖cdot∖text{FANCF}−0.0972∖cdot∖text{CSRNP1}−0.0362∖cdot∖text{NR4A1. The patients were divided into high-risk and low-risk groups based on the median risk score. Using the “survival” package (version 3.3-1) in R, Kaplan-Meier survival analysis was performed to examine the differences in survival between the two groups. The log-rank test was utilized to determine the statistical significance. To further assess the prediction accuracy of the risk model, a time-dependent ROC curve analysis was performed using the “timeROC” package (version 0.4). The area under the ROC curve [24] was utilized to gauge the model’s performance.

### 2.3 Expression Analysis of Six Prognostic Genes in HCC Datasets

Leveraging the Clinical Bioinformatics Home platform, the TCGA-LIHC dataset’s data were obtained and examined (https://www.bioinformatics.com.cn), focusing on the expression patterns of six prognostic genes (*NR4A1*, *CSRNP1*, *FANCF*, *POC5*, *YAE1*, *CENPQ*) in tumor tissues vs. adjacent non-tumor tissues. For validation, datasets GSE36076 and GSE95698 were accessed through the Sangerbox Website (version 3.0, http://vip.sangerbox.com/home.html (accessed on 01 September 2025)). Comparisons were made between the tumor and normal groups for each gene across the three datasets to assess the consistency of expression trends.

### 2.4 Cell Lines and Culture Conditions

The human hepatocyte cell line THLE-2 (CL-0833, Procell Life Science & Technology Co., Ltd., Wuhan, China) was authenticated by short tandem repeat (STR) profiling and confirmed to be free of mycoplasma contamination. The hepatocellular carcinoma (HCC) cell lines used in this study include MHCC97-L (CL-0497), HCCLM3 (CL-0278), Hep 3B2.1-7 (CL-0102), and HuH-7 (CL-0120), all purchased from Procell Life Science & Technology Co., Ltd. STR authentication reports were provided by the supplier and are available on their official product pages. The MHCC97-H cell line was obtained from the Cell Bank of the Chinese Academy of Sciences (Shanghai, China), catalog number SCSP-5082. STR profiling and quality control information, including mycoplasma testing, were provided by the supplier and confirmed before experimental use. All cell lines were confirmed negative for mycoplasma contamination using PCR-based assays (Supplementary Fig. S1). These HCC cells were kept in Dulbecco’s Modified Eagle’s Medium (DMEM, Gibco, Thermo Fisher Scientific, Waltham, MA, USA) at 37°C in a humidified environment with 5% CO_2_, supplemented with 10% fetal bovine serum (FBS; Gibco), 100 U/mL penicillin (Gibco), and 100 µg/mL streptomycin (Gibco).

### 2.5 Cell Transfection and Treatments

Two small interfering RNAs (siRNAs) specifically targeting human *CSRNP1* (designated as si-*CSRNP1*-1 and si-*CSRNP1*-2) and a non-targeting negative control siRNA (si-NC) were synthesized by GenePharma Co., Ltd. (Shanghai, China). For overexpression experiments, the full-length coding sequence of *CSRNP1* was cloned into the pcDNA3.1(+) expression vector to construct the *CSRNP1*-overexpression plasmid, with the corresponding empty vector (pcDNA3.1) used as a control. To achieve 60%–70% confluence, Hep3B and Huh7 cells were seeded into six-well plates at a density of 3 × 10^5^ cells/well and incubated overnight. Following the manufacturer’s instructions, transfections were performed using Lipofectamine™ 3000 (Invitrogen, Thermo Fisher Scientific, Waltham, MA, USA; L3000008) reagent in serum-free Opti-MEM (Invitrogen, USA). SiRNAs (50 nM final concentration) or 2 μg of plasmids were mixed with Lipofectamine 3000 and added to the wells for 6 h, after which the media were replaced with full DMEM containing 10% FBS. Cells were harvested at 48 h post-transfection for subsequent analyses. To investigate ROS-dependent mechanisms, *CSRNP1*-expressing cells were pretreated with 25 mM N-acetylcysteine (NAC; Beyotime, Shanghai, China; S0077), a ROS scavenger, dissolved in Phosphate-Buffered Saline (PBS, Thermo Fisher Scientific, USA) for 48 h. For JNK pathway inhibition, cells were exposed to 10 μM SP600125 (MedChemExpress, Shanghai, China; HY-12041), a specific JNK inhibitor, dissolved in DMSO, for 1 h before subsequent assays.

### 2.6 Quantitative Reverse Transcription Polymerase Chain Reaction (qRT-PCR)

Total RNA was isolated from THLE-2, MHCC97L, HCCLM3, Hep3B, MHCC97H, and Huh7 cells by applying the TRIzol reagent (Thermo Fisher Scientific, USA; 15596026) as directed by the manufacturer, cDNA was synthesized using the PrimeScript RT Reagent Kit (Takara, Japan; RR037A), qRT-PCR was carried out using a StepOnePlus Real-Time PCR System (Applied Biosystems, Thermo Fisher Scientific, USA) and the SYBR Green PCR Master Mix (Takara, Japan), and they were standardized utilizing an internal standard Glyceraldehyde-3-Phosphate Dehydrogenase (*GAPDH*). The expression was measured utilizing the 2^−ΔΔCT^ technique. qRT-PCR primers included: *CSRNP1*: forward 5^′^-GTGGCGTGCCTGTTTGGATG-3^′^, reverse 5^′^-ATCCTGGCGCTGACTCACAG-3^′^; *GAPDH*: forward 5^′^-TCAGCCGCATCTTCTTTTGC-3^′^, reverse 5^′^-ACCTTCCCCATGGTGTCTGA-3^′^.

### 2.7 Western Blotting (WB)

To facilitate the preparation of protein lysates from THLE-2, MHCC97L, HCCLM3, Hep3B, MHCC97H, and Huh7 cells, protease and phosphatase inhibitors (Beyotime, Shanghai, China) were added to the RIPA lysis solution (Beyotime, Shanghai, China). The protein content was determined utilizing the BCA Protein Assay Kit (Beyotime, Shanghai, China; P0010). SDS-PAGE was used to divide the protein into equal portions, which were subsequently transferred onto PVDF membranes (Beyotime, Shanghai, China). Primary antibodies against CSRNP1 (1:1000, Proteintech, Rosemont, IL, USA; 18162-1-AP), Cytochrome C Oxidase Subunits II (COX II, 1:1000, Cell Signaling Technology, Danvers, MA, USA; 31219S), Cytochrome C Oxidase Subunits IV (COX IV, 1:1000, Cell Signaling Technology, 4844S), B-cell lymphoma 2 (Bcl-2, 1:2000, Abcam, Cambridge, UK; ab182858), Poly (ADP-ribose) polymerase 1 (PARP1, 1:1000, Abcam, ab191217), Cleaved PARP1 (1:2000, Abcam, ab32064), p38 MAPK (1:2000, Proteintech, 14064-1-AP), p-p38 MAPK (1:2000, Proteintech, 28796-1-AP), JNK (1:1000, Proteintech, 51153-1-AP), and p-JNK (1:2000, Proteintech, 80024-1-RR) were then used to probe the membranes. For cytoplasmic proteins, GAPDH (1:10000, Abcam, ab181602) was employed as the loading control. Primary antibodies were stored at 4°C overnight. Goat Anti-Rabbit IgG H&L (HRP, 1:2000, Abcam, ab6721) antibodies were incubated for 1 h. Following TBST washing, the protein bands were detected by an ECL detection system (Beyotime, Shanghai, China) and captured on ImageJ software (National Institutes of Health, Bethesda, MD, USA; version 1.8.0).

### 2.8 Cell Counting Kit-8 (CCK-8) Assay

The CCK-8 test was utilized to measure cell viability and proliferation (CK04, Dojindo Laboratories, Kumamoto, Japan). In 96-well plates, Hep3B and Huh7 cells were cultivated at a density of 5 × 10^3^ cells per well. Each well received an addition of CCK-8 reagent at a final concentration of 10% (v/v) following the appropriate procedures. The absorbance was determined utilizing a microplate reader (Multiskan Fc, Thermo Fisher Scientific, Waltham, MA, USA) at 450 nm after 1, 2, 3, and 4 days.

### 2.9 Migration and Invasion Assays

A Transwell device was used to evaluate the Huh7 and Hep3B cells’ capacity for invasion and migration (Costar, Cambridge, UK). A cell suspension in serum-free media was prepared for the migration test. The top chamber of the Transwell insert was filled with 200 μL of cell suspension, containing approximately 5 × 10^4^ cells. To act as a chemoattractant, the bottom compartment was filled with 500 μL of medium that included 10% fetal bovine serum. For 24 h, the cells were kept at 37°C in an incubator with 5% CO_2_. Following incubation, the upper chamber was carefully removed, and a cotton swab was used to gently wipe away any non-migrated cells from the upper surface of the membrane. After that, the migrating cells on the bottom side of the membrane were stained for 15 min at room temperature using a 4^′^,6-diamidino-2-phenylindole (DAPI) solution (1 µg/mL, Thermo Fisher Scientific, USA). Following 1× PBS (pH 7.4) washing, the cells were allowed to air dry before being examined at 200× magnification using an inverted microscope (CKX53, Olympus, Tokyo, Japan). Images were taken for processing, and cell counts were conducted in five randomly selected fields. A Matrigel coating was applied to the membrane of the Transwell insert to simulate the extracellular matrix in the invasion test, which followed a protocol similar to that of the migration assay.

### 2.10 Flow Cytometry

After being plated in 24-well plates at a density of 1 × 10^4^ cells per well, Hep3B and Huh7 cells were cultured for 24 h. Cells were grown with 5 μL of Propidium Iodide (PI) and 5 μL of Annexin V-Fluorescein Isothiocyanate (Annexin V-FITC, Beyotime, Shanghai, China) solution for 15 min to perform an apoptosis assay after being dissociated with trypsin-EDTA (Beyotime, Shanghai, China) and washed with 1× PBS (pH 7.4). The staining of PI and Annexin V was then evaluated using a flow cytometer (CyFlow Space, Jiyuan, Guangzhou, China), and the data were analyzed utilizing FlowJo software (version 10.8, BD Biosciences, Ashland, OR, USA).

### 2.11 Transmission Electron Microscopy (TEM)

Transmission electron microscopy was utilized to investigate ultrastructural alterations in mitochondria. Initially, Hep3B and Huh7 cells were fixed overnight at 4°C in 0.1 M phosphate buffer (pH 7.4) containing 2.5% glutaraldehyde. After the initial fixation, cells were post-fixed for 1 h utilizing 1% osmium tetroxide. Following infiltration and embedding in epoxy resin, the samples were dehydrated using a series of graded ethanols. At an accelerating voltage of 80 kV, ultrathin slices (about 70 nm) were cut using an ultramicrotome (Leica EM UC7, Leica Microsystems, Vienna, Austria), stained with 2% huranyl acetate and 0.4% lead citrate, and examined under a transmission electron microscope (Hitachi HT7700, Hitachi, Tokyo, Japan). To evaluate the structural integrity and morphology of the mitochondria, representative pictures were taken.

### 2.12 Measurement of Reactive Oxygen Species (ROS)

Hep3B and Huh7 cells were cultivated at a density of 1 × 10^4^ cells per well in 96-well plates. Following cell adhesion, the monolayers underwent two 1× PBS (pH 7.4) washes before being lysed. The resulting lysates were centrifuged for 5 min at 10,000× *g* to extract the supernatant. These supernatants underwent sonication using a Scientz-IID ultrasonic homogenizer (Scientz, Ningbo, China), and the levels of ROS were then measured. Commercial assay kits (E004-1-1, Nanjing Jiancheng Bioengineering Institute, Nanjing, China) were used to make measurements as directed by the manufacturer.

### 2.13 Assessment of Intracellular and Mitochondrial ROS (mROS)

Hep3B and Huh7 cells were transfected to overexpress *CSRNP1* and treated for 48 h to measure the levels of intracellular and mitochondrial ROS. DCFH-DA (Sigma-Aldrich, St. Louis, MO, USA) was used to assess intracellular ROS levels, and MitoSOX™ Red mitochondrial superoxide indicator (Invitrogen, USA) was used to measure Mitochondrial Reactive Oxygen Species (mROS). To detect total ROS, cells were treated with 10 μM DCFH-DA at 37°C for 30 min in the dark. Cells were treated with 5 μM MitoSOX™ Red under the same conditions to detect mROS. Following staining, cells were immediately examined under a fluorescent microscope (Leica DMi8, Leica Microsystems, Wetzlar, Germany) and rinsed three times with PBS. Green fluorescence (DCFH-DA) indicated intracellular ROS, while red fluorescence (MitoSOX Red) indicated mitochondrial superoxide production. Quantitative analysis was performed using ImageJ software based on fluorescence intensity.

### 2.14 Statistical Analysis

For the statistical analysis, the R software was used. Each experiment was used three times, and the findings were described using the mean ± SD. A one-way ANOVA was used to evaluate the significant differences across multiple groups. Pairwise comparisons were conducted using Tukey’s post hoc test. Two-way ANOVA was used for analyses with more than one variable, and post-hoc tests were adjusted using Bonferroni correction for multiple comparisons. The *p*-value was considered statistically significant if it was less than 0.05.

## 3 Results

### 3.1 Cross-Dataset Identification of Overlapping DEGs in HCC

**Figure 1:**
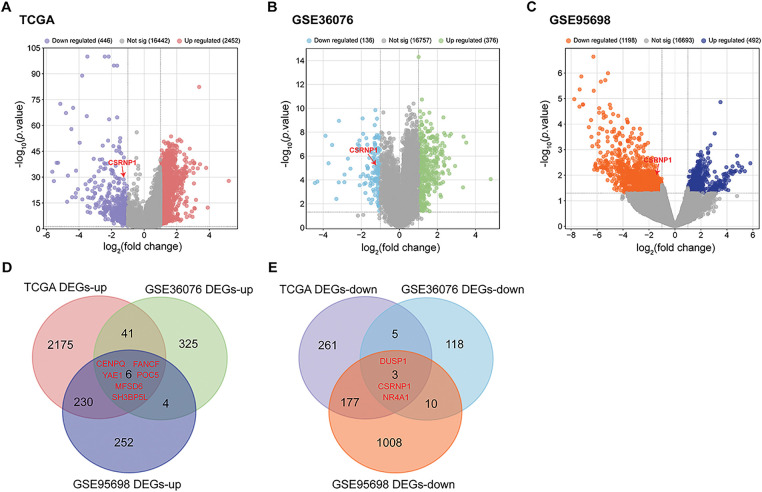
Integrated analysis reveals DEGs across multiple HCC datasets. (**A**) Volcano plots showing DEGs in the TCGA-LIHC dataset. Red dots indicate significantly upregulated DEGs; blue dots represent significantly downregulated DEGs. (**B**) Volcano plots showing DEGs in the GSE36076 dataset. Green dots indicate significantly upregulated DEGs; light blue dots represent significantly downregulated DEGs. (**C**) Volcano plots showing DEGs in the GSE95698 dataset. Dark blue dots indicate significantly upregulated DEGs; orange dots represent significantly downregulated DEGs. (**D**) Venn diagram of upregulated DEGs from the three datasets, highlighting six common upregulated genes. (**E**) Venn diagram of downregulated DEGs, identifying three overlapping downregulated genes. HCC: Hepatocellular Carcinoma; DEGs: Differentially Expressed Genes

Differential expression analysis was conducted across three HCC-related datasets to identify DEGs. The TCGA-LIHC dataset identified 2452 upregulated DEGs and 446 downregulated DEGs. Analysis of the GSE36076 dataset revealed 376 upregulated and 136 downregulated DEGs, while the GSE95698 dataset yielded 492 upregulated and 1198 downregulated DEGs (Fig. 1A–C). Through Venn’s analysis, we identified a total of 6 up-regulated DEGs in all three datasets, namely *CENPQ*, *POC5*, *YAE1*, *FANCF*, *MFSD6*, and *SH3BP5L*, as well as 3 down-regulated differentially expressed genes, namely *DUSP1*, *NR4A1*, and *CSRNP1* (Fig. 1D,E). These nine overlapping genes show their potential as central regulators in hepatocarcinogenesis.

### 3.2 Prognostic Signature Construction Using LASSO Cox Regression Identifies a Six-Gene Risk Model in HCC

**Figure 2:**
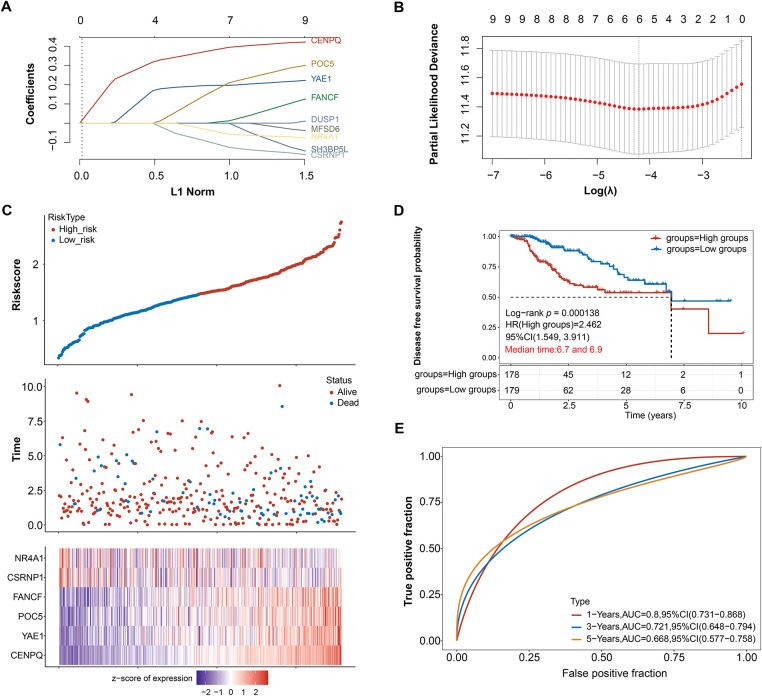
Prognostic significance of candidate genes in HCC. (**A**) The coefficient spectrum obtained through LASSO regression analysis for 9 potential prognostic genes. Each line represents a gene. (**B**) The LASSO regression was performed with tenfold cross-validation to determine the optimal lambda value. Red dots represent partial likelihood deviance, and the gray line indicates the standard error. (**C**) The upper panel displays risk score curves for low and high-risk groups, the middle panel depicts patient survival status, and the lower panel presents the heatmap of expression profiles for the four prognostic genes. (**D**) The survival curves represent Kaplan-Meier analysis for the six prognostic genes. The *x*-axis denotes time in years, and the *y*-axis indicates disease-free survival probability. (**E**) ROC curves of the risk model in 1, 3, and 5 years. The curves in different colors represent the AUC values at various periods. HCC: Hepatocellular Carcinoma; LASSO: Least Absolute Shrinkage and Selection Operator; ROC: Receiver Operating Characteristic; AUC: Area Under the Curve; HR: High Groups

To identify prognostic biomarkers in HCC, LASSO Cox regression analysis was combined with 10-fold cross-validation. At the optimal lambda value (λ_min = 0.0149), six candidate genes were selected as the most robust prognostic features (Fig. 2A,B). A risk score was determined for every patient based on the expression patterns of these genes (*NR4A1*, *CSRNP1*, *FANCF*, *POC5*, *YAE1*, and *CENPQ*). The median risk score was then used as the cutoff to stratify the patients into high-risk and low-risk groups. A heatmap displaying the distribution of survival outcomes and the expression patterns of the six-gene signature shown in Fig. 2C. Patients in the high-risk group had a significantly lower disease-free survival probability compared to those in the low-risk group (Log-rank *p* = 0.000138, HR = 2.462, 95% CI = 1.548–3.911), as determined by Kaplan-Meier survival analysis (Fig. 2D). The risk model’s predictive ability was demonstrated through time-dependent ROC curve analysis, with an AUC of 0.800 (95% CI = 0.731–0.800) at 1 year, 0.721 (95% CI = 0.648–0.794) at 3 years, and 0.668 (95% CI = 0.577–0.758) at 5 years, indicating strong prognostic performance (Fig. 2E).

### 3.3 Expression Profiling Highlights CSRNP1 as a Potential Hub Gene in HCC

**Figure 3:**
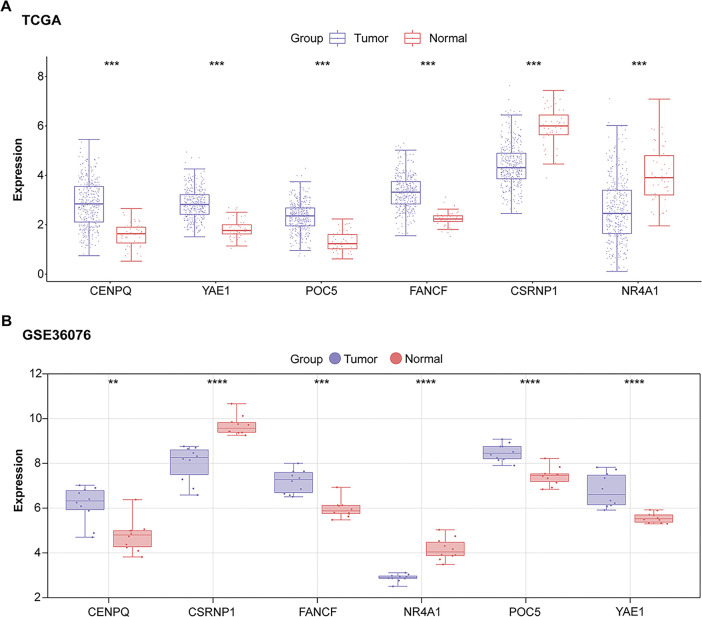

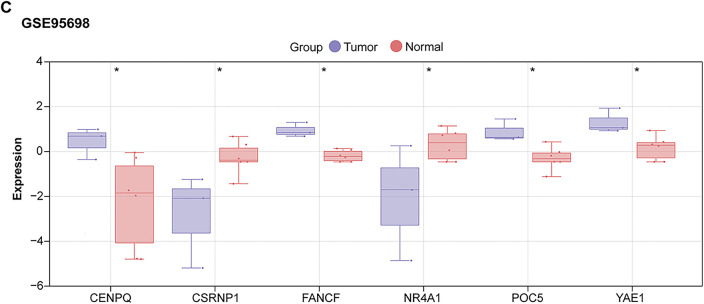
Expression analysis of prognostic genes identifies *CSRNP1* as a candidate hub gene in HCC. (**A**) Box plots showing the expression levels of six prognostic genes (*NR4A1*, *CSRNP1*, *FANCF*, *POC5*, *YAE1*, and *CENPQ*) in tumor vs. normal liver tissues from the TCGA-LIHC dataset. (**B**) Box plots showing the expression levels of six prognostic genes in tumor vs. normal liver tissues from the GSE36076 dataset. (**C**) Box plots showing the expression levels of six prognostic genes in tumor vs. normal liver tissues from the GSE95698 dataset. TCGA: The Cancer Genome Atlas; HCC: Hepatocellular Carcinoma. *p < 0.05 or **p < 0.01 or ***p < 0.001 or ****p < 0.0001 vs. Normal group

To investigate the clinical relevance of the six prognostic genes, their expression levels were analyzed in datasets related to HCC. When comparing tumor tissues to standard controls, a substantial upregulation of four genes was observed: *CENPQ*, *YAE1*, *FANCF*, and *POC5*. In contrast, *NR4A1* and *CSRNP1* exhibited markedly lower expression in the tumor group (Fig. 3A–C). Notably, *CSRNP1* has been identified as a significant prognostic marker in HCC based on previous bioinformatics analyses; however, its precise biological functions and underlying mechanisms in hepatocarcinogenesis remain unclear, indicating substantial potential for further investigation.

### 3.4 Downregulation of CSRNP1 in HCC Cell Lines and Validation of siRNA-Mediated Knockdown Efficiency

**Figure 4:**
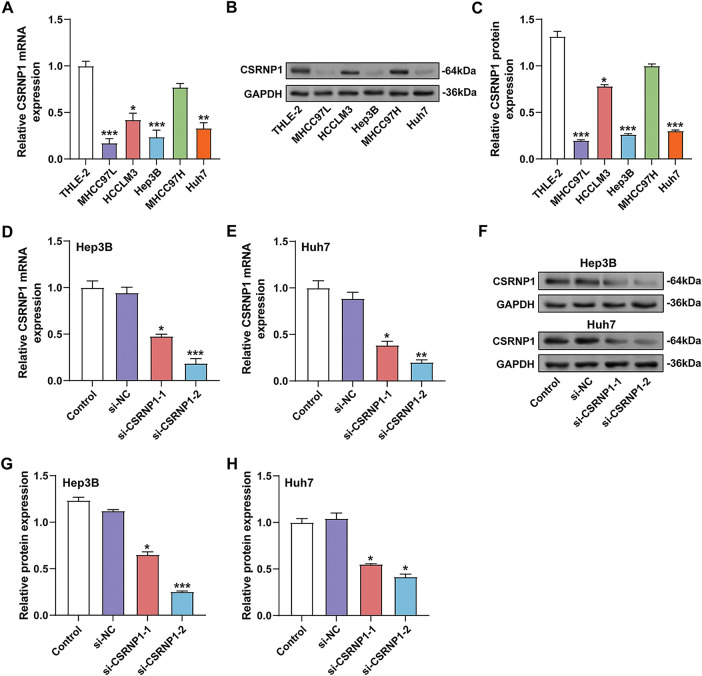
*CSRNP1* is downregulated in HCC cell lines and efficiently silenced by siRNA transfection. (**A**) qRT-PCR analysis of *CSRNP1* mRNA expression in normal hepatic epithelial cells (THLE-2) and five HCC cell lines (MHCC97L, HCCLM3, Hep3B, MHCC97, and Huh7). *p < 0.05 or **p < 0.01 or ***p < 0.001 vs. THLE-2 group. (**B**,**C**) WB analysis of *CSRNP1* protein levels across the same cell lines. *p < 0.05 or ***p < 0.001 vs. THLE-2 group. (**D**,**E**) qRT-PCR showing knockdown efficiency of two siRNAs (si-*CSRNP1*-1 and si-*CSRNP1*-2) targeting *CSRNP1* in Hep3B (**D**) and Huh7 (**E**) cells. *p < 0.05 or **p < 0.01 or ***p < 0.001 vs. si-NC group. (**F**) WB validation of *CSRNP1* protein knockdown in Hep3B and Huh7 cells. (**G**,**H**) Quantification of WB results demonstrates that si-*CSRNP1*-1 and si-*CSRNP1*-2 achieve silencing efficiency in both cell lines. *p < 0.05 or ***p < 0.001 vs. si-NC group. HCC: Hepatocellular Carcinoma; qRT-PCR: Quantitative Reverse Transcription Polymerase Chain Reaction; WB: Western Blotting

Using qRT-PCR and WB analysis, CSRNP1 expression was found to be significantly higher in normal hepatic epithelial cells (THLE-2) than in a variety of HCC cell lines (including MHCC97L, HCCLM3, Hep3B, MHCC97, and Huh7) (Fig. 4A–C). Among them, *CSRNP1* mRNA levels were decreased by about 75% and 60% in Hep3B and Huh7, respectively, and protein expression was reduced by about 75% and 65%, respectively, suggesting that *CSRNP1* may have oncostatic functions. Given the broad application of Hep3B and Huh7 in HCC studies, we selected these two cells for subsequent mechanistic studies. To verify the knockdown efficiency of *CSRNP1*, we transfected two siRNAs (si-*CSRNP1*-1 and si-*CSRNP1*-2) into Hep3B and Huh7 cells. qRT-PCR and WB assays showed that both siRNAs significantly reduced CSRNP1 expression. In Hep3B cells, si-*CSRNP1*-1 and si-*CSRNP1*-2 decreased mRNA levels by about 50% and 75%, and protein levels by about 40% and 75%, respectively; in Huh7 cells, the decreases were about 50% and 70% (mRNA) and 40% and 60% (protein), respectively (Fig. 4D–H). Due to the higher efficiency of si-*CSRNP1*-2, it was selected for subsequent functional experiments.

### 3.5 CSRNP1 Knockdown Enhances the Proliferation, Invasion, and Migration of HCC Cells

**Figure 5:**
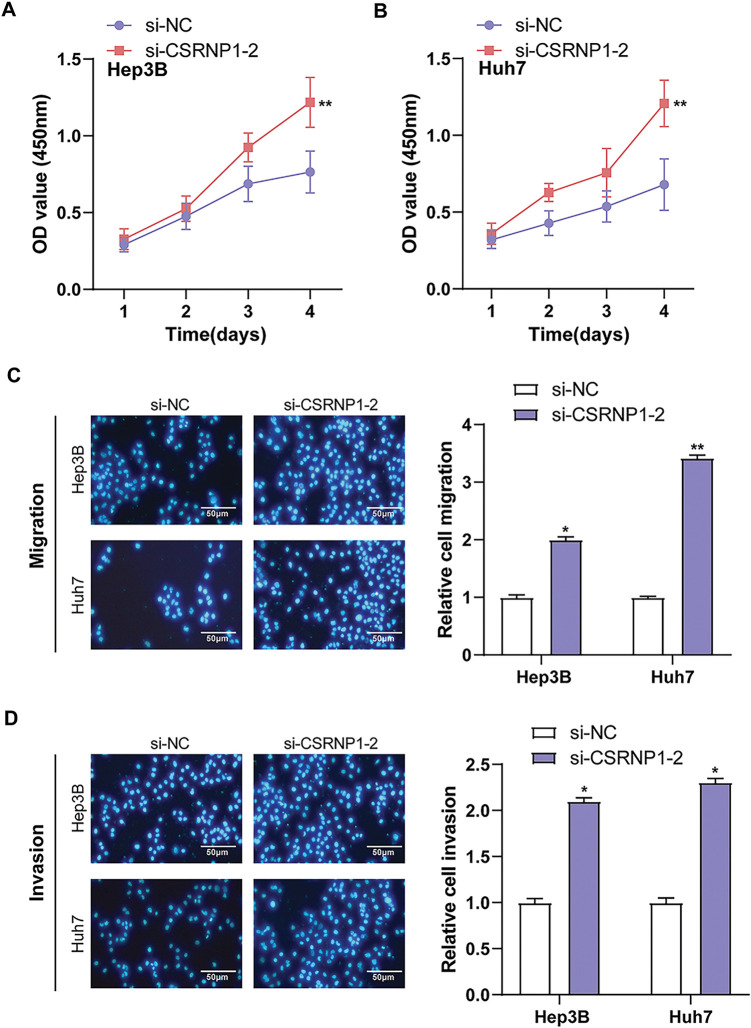
*CSRNP1* knockdown promotes proliferation, migration, and invasion in HCC cells. (**A**,**B**) CCK-8 assays assessing the proliferation of Hep3B (**A**) and Huh7 (**B**) cells following transfection with si-*CSRNP1*-2 or si-NC (negative control). (**C**,**D**) Transwell assays evaluating the effects of *CSRNP1* knockdown on the migration and invasion abilities of Hep3B (**C**) and Huh7 (**D**) cells. Migrated or invaded cells were stained and counted under a microscope. Magnification: 200×, scale bar = 50 μm. HCC: Hepatocellular Carcinoma; CCK-8: Cell Counting Kit-8. *p < 0.05 or **p < 0.01 vs. si-NC group

To investigate the functional role of *CSRNP1* in hepatocellular carcinoma, we evaluated the effects of its knockdown on cell proliferation, migration, and invasion. The CCK-8 assay showed that knockdown of *CSRNP1* significantly enhanced the proliferative ability of Hep3B and Huh7 cells. On day 4, the OD values of Hep3B and Huh7 were increased from approximately 0.75 and 0.7 in the control group to approximately 1.2 in the si-*CSRNP1*-2 group, respectively (Fig. 5A,B). The Transwell assay further confirmed that knockdown of *CSRNP1* significantly promoted cell migration and invasion. Hep3B cells showed a 1-fold increase in migration and invasion, while Huh7 cells showed a 2.4- and 1.25-fold increase in migration and invasion, respectively (Fig. 5C,D). Taken together, *CSRNP1* may play a negative regulatory role in HCC by inhibiting proliferation and motility.

### 3.6 CSRNP1 Knockdown Suppresses Apoptosis in HCC Cells

**Figure 6:**
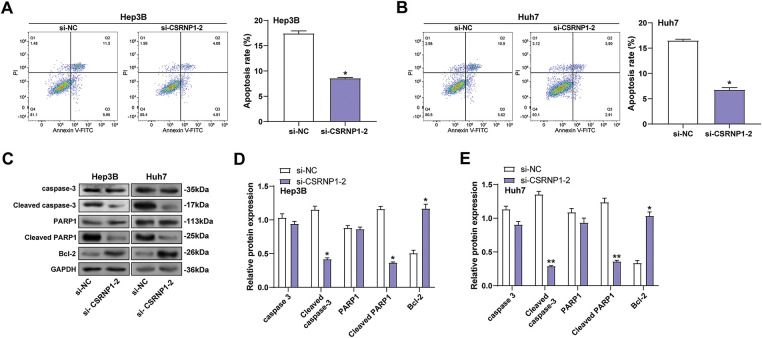
*CSRNP1* knockdown inhibits apoptosis in HCC cells by modulating apoptosis-related protein expression. (**A**,**B**) Flow cytometry analysis of apoptosis in Hep3B (**A**) and Huh7 (**B**) cells after transfection with si-*CSRNP1*-2 or si-NC. The *x*-axis represents Annexin V-FITC fluorescence, and the *y*-axis represents PI fluorescence. Quantification of total apoptotic cells is shown as bar graphs. (**C**) WB analysis of apoptosis-related proteins (caspase-3, cleaved caspase-3, PARP1, cleaved PARP1, and Bcl-2) in Hep3B and Huh7 cells following *CSRNP1* knockdown. (**D**,**E**) Quantification of the indicated protein bands. HCC: Hepatocellular Carcinoma; WB: Western Blotting; PI: Propidium Iodide. *p < 0.05 or **p < 0.01 vs. si-NC group

To evaluate the effect of *CSRNP1* knockdown on apoptosis in HCC cells, we found that *CSRNP1* knockdown significantly inhibited apoptosis in Hep3B and Huh7 cells, as determined by flow cytometry analysis, with apoptosis rates decreasing by approximately 55% and 60%, respectively (Fig. 6A,B). WB further detected changes in the expression of proteins related to apoptosis. After knocking down *CSRNP1*, the anti-apoptotic protein Bcl-2 was elevated about 2- and 1.5-fold in Hep3B and Huh7 cells, respectively (Fig. 6D,E). In contrast, the pro-apoptotic proteins cleaved caspase-3 and cleaved PARP1 were significantly downregulated, by approximately 65% in Hep3B cells and 80% and 75% in Huh7 cells, respectively (Fig. 6C–E). In conclusion, *CSRNP1* may play a pro-apoptotic role in HCC by inhibiting the up-regulation of Bcl-2 and weakening the caspase-dependent apoptotic pathway.

### 3.7 CSRNP1 Overexpression Impairs Mitochondrial Integrity and Enhances ROS Accumulation in HCC Cells

**Figure 7:**
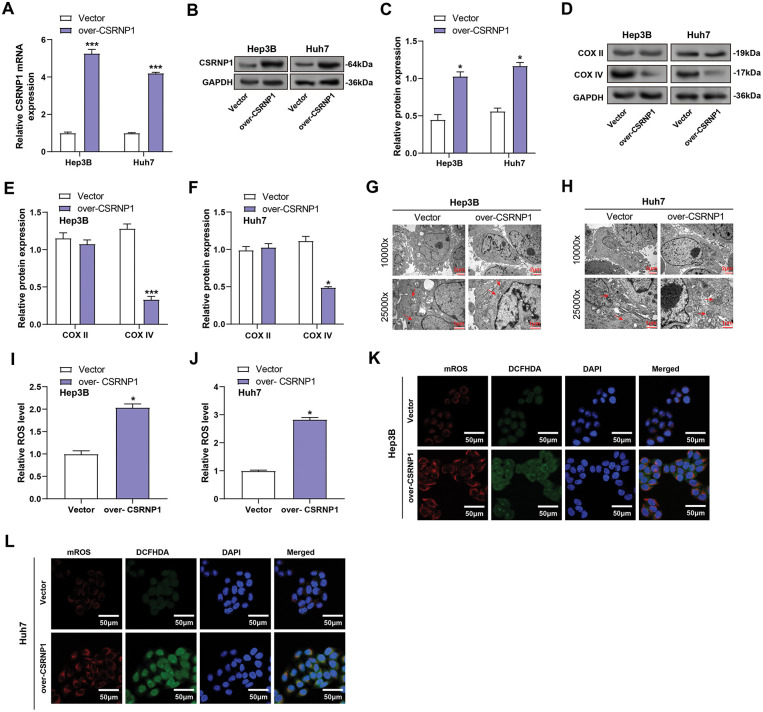
*CSRNP1* overexpression disrupts mitochondrial integrity and increases ROS accumulation in HCC cells. (**A**–**C**) qRT-PCR analyses assess *CSRNP1* overexpression in Hep3B and Huh7 cells after plasmid transfection. (**B**,**C**) WB analyses assess *CSRNP1* overexpression in Hep3B and Huh7 cells after plasmid transfection. Quantitative data (**C**) represent relative protein expression normalized. (**D**–**F**) WB and quantification (**E**,**F**) of mitochondrial respiratory chain complex proteins COX II and COX IV in *CSRNP1*-overexpressing Hep3B and Huh7 cells. (**G**,**H**) TEM images showing mitochondrial ultrastructure in Hep3B (**G**) and Huh7 (**H**) cells. Scale bars: main image = 2 μm, inset = 1 μm. (**I**,**J**) Quantification of total ROS using a ROS detection assay. The *x*-axis indicates treatment group (Vector vs. over-*CSRNP1*), and the *y*-axis indicates relative ROS levels. (**K**,**L**) Assessment of cytoplasmic ROS and mROS levels using specific fluorescent probes. HCC: Hepatocellular Carcinoma; qRT-PCR: Quantitative Reverse Transcription Polymerase Chain Reaction; WB: Western Blotting; TEM: Transmission Electron Microscopy; ROS: Reactive Oxygen Species. *p < 0.05 or ***p < 0.001 vs. Vector group

After transfection of the *CSRNP1* expression plasmid, qRT-PCR and WB results confirmed that its expression was significantly up-regulated in Hep3B and Huh7 cells, with mRNA increasing about 5- and 3-fold, and protein levels increasing about 1- and 1.4-fold, respectively (Fig. 7A–C). Subsequently, its effect on mitochondrial function-related proteins was assessed, and WB showed that COX IV expression was significantly down-regulated, with a decrease of about 65% and 55% in Hep3B and Huh7, respectively. At the same time, COX II levels did not change significantly (Fig. 7D–F). Transmission electron microscopy revealed that *CSRNP1* overexpression led to mitochondrial swelling and disruption of the cristae in both cell lines, indicating damage to the mitochondrial structure (Fig. 7G,H). Further analysis revealed that *CSRNP1* overexpression significantly increased total intracellular ROS by approximately 1- and 1.8-fold in Hep3B and Huh7, respectively (Fig. 7I,J). Both cytoplasmic ROS and mROS levels were significantly elevated (Fig. 7K,L), suggesting that *CSRNP1* overexpression could disrupt mitochondrial homeostasis and induce oxidative stress in HCC cells.

### 3.8 CSRNP1 Activates the JNK/p38 MAPK Pathway through ROS Accumulation in HCC Cells

**Figure 8:**
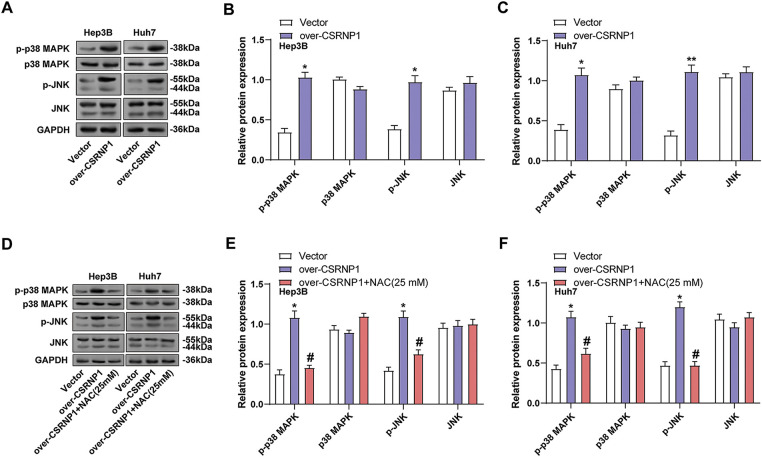
*CSRNP1* activates the JNK/p38 MAPK pathway via ROS accumulation in HCC cells. (**A**–**C**) WB analysis of JNK/p38 MAPK pathway-related proteins in Hep3B and Huh7 cells transfected with *CSRNP1* overexpression vector or control. *p < 0.05 or **p < 0.01 vs. Vector group. (**D**–**F**) WB analysis of JNK/p38 MAPK pathway-related proteins in *CSRNP1*-overexpressing cells treated with NAC. *p < 0.05 vs. Vector group. ^#^*p* < 0.05 vs. Over-*CSRNP1* group. HCC: Hepatocellular Carcinoma; WB: Western Blotting; ROS: Reactive Oxygen Species

To further explore the mechanism of action of *CSRNP1*, we assessed the activation of the JNK/p38 MAPK pathway in Hep3B and Huh7 cells overexpressing *CSRNP1*. WB results showed that *CSRNP1* overexpression significantly increased the levels of p-JNK and p-p38 MAPK, while total protein levels remained unchanged, indicating that the MAPK signaling was activated (Fig. 8A–C). Specifically, in Hep3B cells, p-JNK and p-p38 were upregulated by approximately 1.5- and 1.25-fold, respectively. In contrast, in Huh7 cells, they were upregulated by approximately 1.75- and 2.6-fold, respectively. To verify whether the activation of this pathway was dependent on ROS, cells treated with NAC showed a significant decrease in p-JNK and p-p38 expression (Fig. 8D–F). Specifically, in Hep3B cells, there was approximately a 40% and 60% downregulation for p-JNK and p-p38, respectively, while in Huh7 cells, there was roughly a 60% and 40% downregulation, respectively. These results support that *CSRNP1* activates the JNK/p38 MAPK pathway through an ROS-dependent mechanism, linking oxidative stress with the stress signaling network in HCC.

### 3.9 CSRNP1 Overexpression Activates JNK/p38 MAPK Pathway and Induces Apoptosis in HCC Cells

**Figure 9:**
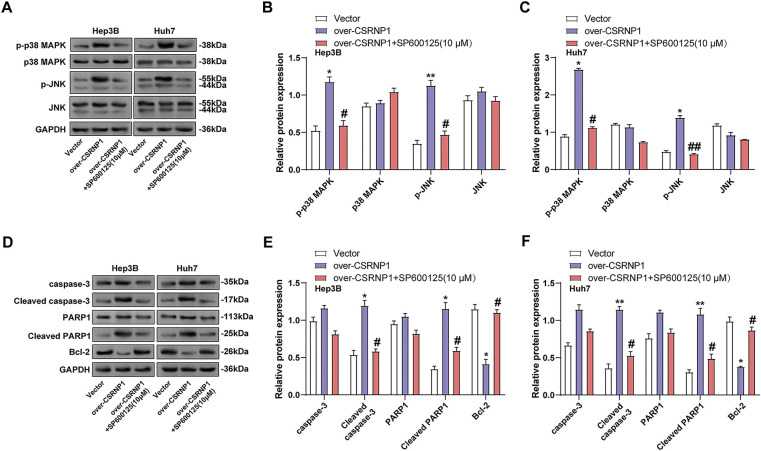
*CSRNP1* promotes apoptosis in HCC cells via JNK/p38 MAPK pathway activation. (**A**–**C**) WB analysis showing levels of JNK/p38 MAPK pathway-related proteins following *CSRNP1* overexpression with or without SP600125 treatment. (**D**–**F**) Apoptosis-related protein expression was assessed following *CSRNP1* overexpression with or without SP600125 treatment. *p < 0.05 or **p < 0.01 vs. Vector group. ^#^*p* < 0.05 or ^##^*p* < 0.05 vs. Over-*CSRNP1* group

To verify whether *CSRNP1*-induced signaling was dependent on the JNK/p38 MAPK pathway, we combined the JNK inhibitor SP600125 in *CSRNP1*-overexpressing Hep3B and Huh7 cells and performed WB analysis. The results showed that *CSRNP1* significantly upregulated p-p38 and p-JNK levels, which were elevated about 1.4- and 1.75-fold in Hep3B cells, and about 2.1- and 3.8-fold in Huh7, respectively (Fig. 9A–C). Combined SP600125 treatment restored these phosphorylation levels to near baseline, suggesting that MAPK signaling activation is dependent on the JNK pathway. Further analysis revealed that *CSRNP1* overexpression significantly upregulated cleaved PARP1 and cleaved caspase-3 and downregulated Bcl-2, suggesting that it promotes apoptosis. In Hep3B, cleaved caspase-3 and cleaved PARP1 were up-regulated by about 1.4- and 2.6-fold, respectively, and Bcl-2 decreased by about 60%; the corresponding changes in Huh7 were 2.6-, 3.4-fold, and 60%, respectively (Fig. 9D–F). The above effects were almost completely reversed after SP600125 co-treatment, further supporting the notion that CSRNP1 induces apoptosis in HCC cells through the activation of the JNK/p38 MAPK pathway.

## 4 Discussion

Tumor heterogeneity poses substantial challenges to the diagnosis and treatment of HCC, which remains a leading cause of cancer-related mortality worldwide [25]. As such, extensive efforts have been made to identify biomarkers that facilitate early detection, accurate diagnosis, and personalized therapeutic strategies. In our analysis, differential gene expression screening across three datasets identified nine overlapping DEGs, from which a six-gene risk signature—comprising *CENPQ*, *YAE1*, *POC5*, *FANCF*, *CSRNP1*, and *NR4A1*—was constructed via LASSO regression. Prior studies have reported that *CENPQ* is upregulated in HCC, promoting proliferation and influencing a poor prognosis by affecting immune infiltration and cell cycle progression [26]. *NR4A1* expression is suppressed by the HCV core protein, reducing the sensitivity of HCC cells to cisplatin and impairing apoptosis, which may contribute to HCV-related hepatocarcinogenesis [27]. The roles of *YAE1*, *POC5*, and *FANCF* in HCC remain largely unexplored. While *CSRNP1* has been highlighted in HCC and other cancers through bioinformatic analyses [16,17], its functional contribution to HCC development has not been well characterized. In this study, we confirmed that *CSRNP1* is downregulated in HCC cell lines. Functional assays further revealed that *CSRNP1* knockdown enhanced HCC cell growth and invasiveness while suppressing apoptosis, with upregulation of Bcl-2 and reduced expression of cleaved caspase-3 and cleaved PARP1. These results support a tumor-suppressive role for *CSRNP1* in HCC, potentially mediated through regulation of apoptosis.

Mitochondrial dysfunction is frequently observed in cells exposed to oxidative stress, DNA damage, or calcium overload [28]. COX IV, a key subunit of cytochrome c oxidase, is essential for ATP production in the mitochondrial respiratory chain [29]. Studies have shown that XRCC2 deficiency leads to the accumulation of DNA damage at replication sites, impairing mitochondrial respiration and reducing complex I activity in HCC cells [30]. Similarly, Donafenib induces oxidative stress in HCC cells by increasing mROS, decreasing GPx activity, and suppressing Mn-SOD, ultimately reducing mitochondrial membrane potential, COX IV activity, and ATP levels [31]. Osteopontin (OPN) has been reported to promote HCC cell proliferation and migration via ROS generation and JAK2/STAT3-mediated NOX1 upregulation, establishing a positive feedback loop [32]. In our study, CSRNP1 overexpression led to apparent mitochondrial structural damage, including cristae disruption and swelling, accompanied by a decrease in COX IV levels. Moreover, *CSRNP1* induced a significant increase in intracellular ROS, suggesting that *CSRNP1* contributes to mitochondrial dysfunction and oxidative stress in HCC cells.

ROS are key upstream activators of the JNK/p38 MAPK pathway, initiating stress response signaling cascades. In turn, JNK/p38 MAPK signaling can modulate ROS homeostasis by regulating both oxidative and antioxidative systems [33,34]. To elucidate the interplay between *CSRNP1*, ROS, and MAPK signaling, we employed two pharmacological agents: NAC, a broad-spectrum ROS scavenger [35], and SP600125, a selective ATP-competitive inhibitor of JNK [36]. Prior studies support this framework: Echinatin induces apoptosis in colorectal cancer cells by triggering ROS accumulation and activating the JNK/p38 MAPK pathway, with ROS inhibition mitigating these effects [37]. Resistomycin also promotes apoptosis and cell cycle arrest in HCC via p38 MAPK activation, which can be reversed by p38-specific inhibitors [38]. GLUD1, downregulated in HCC, activates the JNK/p38 MAPK pathway through enhanced ROS production and mitochondrial respiration, suppressing tumor progression—a process reversible by ROS scavengers [39]. In our investigation, *CSRNP1* overexpression increased the phosphorylation levels of JNK and p38 MAPK, which NAC reduced. Furthermore, SP600125 reversed the pro-apoptotic effects of CSRNP1, as evidenced by increased cleaved caspase-3 and PARP1, and decreased Bcl-2 expression. These results indicate that *CSRNP1*-induced apoptosis in HCC is mediated via a ROS-dependent JNK/p38 MAPK signaling axis.

It is essential to acknowledge that MAPK pathways—including JNK, p38, and ERK1/2—can exert either pro-apoptotic or pro-survival effects, depending on their activation dynamics, cellular context, and interactions with other signaling networks [40]. In our study, *CSRNP1* predominantly activated the JNK/p38 pathway in a ROS-dependent manner, leading to apoptosis. However, it remains unclear whether *CSRNP1* also modulates other pro-survival pathways. Notably, the PI3K/AKT and NF-κB pathways are key survival-promoting cascades in HCC. Oxidative stress and JNK activation are known to negatively regulate AKT signaling, thereby tipping the balance toward apoptosis. In addition, ERK1/2 is frequently associated with cell proliferation, survival, and metastasis in HCC [41]. It is therefore plausible that *CSRNP1* may antagonize ERK1/2 signaling as part of its tumor-suppressive function. Future studies employing phosphoproteomics and specific pathway inhibitors will be critical for mapping the broader signaling networks regulated by *CSRNP1* and delineating its interactions with cellular stress and survival pathways.

Despite the robust evidence presented, this study has several limitations. First, although *CSRNP1* was confirmed as a downregulated gene in HCC via integrated transcriptomic analysis and validated in cell lines, we did not perform immunohistochemical (IHC) validation using clinical tissue samples. The lack of IHC data limits the translational relevance of our findings and precludes a spatial assessment of *CSRNP1* expression in the tumor microenvironment. Future studies incorporating IHC on clinical tissue arrays are necessary to clarify the diagnostic and prognostic potential of *CSRNP1*. Second, our mechanistic investigations were confined to *in vitro* models. Although informative, these do not fully replicate the complexity of the *in vivo* tumor context. Follow-up studies using xenograft or orthotopic mouse models are crucial for evaluating the systemic effects of *CSRNP1* on tumor growth, apoptosis, and metastasis. Third, while we established the ROS-dependent JNK/p38 MAPK activation as a key mechanism underlying *CSRNP1*-induced apoptosis, additional downstream effectors and regulatory feedback loops remain to be defined. Comprehensive transcriptomic and phosphoproteomic profiling following *CSRNP1* perturbation would provide further insight into the breadth of its regulatory functions.

In conclusion, our findings demonstrate that *CSRNP1* functions as a tumor suppressor in HCC, exerting its anti-tumor effects through the ROS-dependent activation of the JNK/p38 MAPK signaling pathway. These results underscore the potential of *CSRNP1* as both a prognostic biomarker and a therapeutic target in HCC. Further validation in clinical samples and *in vivo* models is warranted to facilitate the translation of this approach into clinical practice.

## 5 Conclusion

Our findings demonstrate that *CSRNP1* functions as a tumor suppressor in HCC by regulating the ROS-mediated activation of the JNK/p38 MAPK pathway, leading to mitochondrial dysfunction and apoptosis. Its downregulation in HCC tissues, along with a negative association with malignant phenotypes, underscores its critical role in restraining tumor progression. Overexpression of *CSRNP1* leads to increased ROS production, activation of the JNK/p38 MAPK pathway, and mitochondrial damage, ultimately promoting apoptosis in HCC cells. These results identify *CSRNP1* as a key upstream modulator of the ROS–MAPK signaling axis, highlighting its potential as both a prognostic biomarker and a therapeutic target in HCC.

## Supplementary Materials

Figure S1 Detection of Mycoplasma contamination in cultured cell lines by PCR-based assay. Agarose gel electrophoresis showing PCR products of mycoplasma-specific DNA in various cell lines and controls. Lanes include: DNA marker (leftmost lane), negative control, six independent positive controls (Positive control 1-6), and the tested cell lines THLE-2, MHCC97L, HCCLM3, Hep3B, MHCC97H, and Huh7. No amplification bands were observed in any of the tested cell lines, consistent with the negative control, indicating the absence of mycoplasma contamination. The presence of bands in positive control lanes validates assay performance.

## Data Availability

The datasets used and/or analyzed during the current study are available from the corresponding authors upon reasonable request.

## Supplementary Materials

The supplementary material is available online at https://www.techscience.com/doi/10.32604/or.2025.068737/s1.
