# Supplementary figures and images for: CSRNP1 Promotes Apoptosis and Mitochondrial Dysfunction via ROS-Mediated JNK/p38 MAPK Pathway Activation in Hepatocellular Carcinoma
Source: Oncol Res. 2025 Dec 30;34(1):17. doi: 10.32604/or.2025.068737 (PMC12774537; doi:10.32604/or.2025.068737)

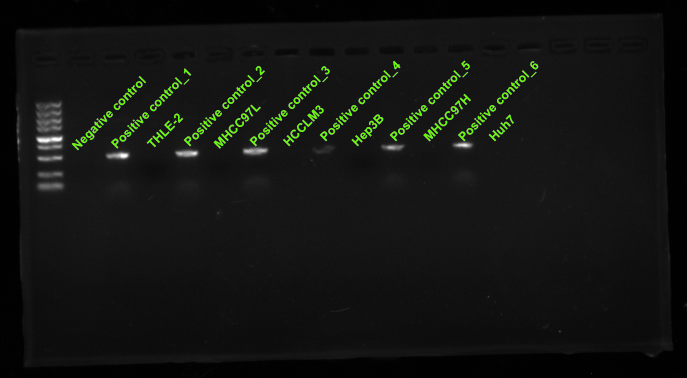

Supplement: Figure S1 [file OncolRes-34-68737-s001.tif]
