# Supplementary material for: CSRNP1 Promotes Apoptosis and Mitochondrial Dysfunction via ROS-Mediated JNK/p38 MAPK Pathway Activation in Hepatocellular Carcinoma
Source: Oncol Res. 2025 Dec 30;34(1):17. doi: 10.32604/or.2025.068737 (PMC12774537; doi:10.32604/or.2025.068737)
Supplement: Supplementary file 2 [file OncolRes-34-68737-s002.docx]

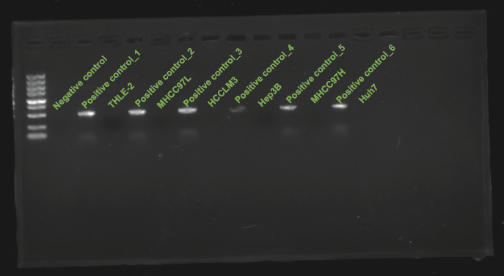


**Supplementary Figure 1. Detection of Mycoplasma contamination in cultured cell lines by PCR-based assay.** Agarose gel electrophoresis showing PCR products of mycoplasma-specific DNA in various cell lines and controls. Lanes include: DNA marker (leftmost lane), negative control, six independent positive controls (Positive control 1-6), and the tested cell lines THLE-2, MHCC97L, HCCLM3, Hep3B, MHCC97H, and Huh7. No amplification bands were observed in any of the tested cell lines, consistent with the negative control, indicating the absence of mycoplasma contamination. The presence of bands in positive control lanes validates assay performance.
